# Protopanaxadiol inhibits epithelial–mesenchymal transition of hepatocellular carcinoma by targeting STAT3 pathway

**DOI:** 10.1038/s41419-019-1733-8

**Published:** 2019-08-20

**Authors:** Lan Yang, Xue-ying Zhang, Kun Li, An-ping Li, Wen-dong Yang, Ru Yang, Peng Wang, Zi-han Zhao, Fang Cui, Yuan Qin, Jia-huan Yang, Hong-lian Tao, Tao Sun, Shuang Chen, Pei-hua Yu, Hui-juan Liu, Cheng Yang

**Affiliations:** 1 State Key Laboratory of Medicinal Chemical Biology and College of Pharmacy, Nankai University, Tianjin, China; 2 Tianjin Key Laboratory of Early Druggability Evaluation of Innovative Drugs and Tianjin Key Laboratory of Molecular Drug Research, Tianjin International Joint Academy of Biomedicine, Tianjin, China; 3 College of Life Sciences, Nankai University, Tianjin, China; 4 College of Marine and Environmental Sciences, Tianjin University of Science and Technology, Tianjin, China; 5 Enoch Phytomedicine Ltd., Shenzhen, China

**Keywords:** Metastasis, Cell migration

## Abstract

Diol-type ginsenosides, such as protopanaxadiol (PPD), exhibit antioxidation, anti-inflammation, and antitumor effects. However, the antitumor effect of these ginsenosides and the mechanism of PPD remain unclear. In this work, the antitumor effects of several derivatives, including PPD, Rg5, Rg3, Rh2, and Rh3, were evaluated in five different cancer cell lines. PPD demonstrated the best inhibitory effects on the proliferation and migration of the five cancer cell lines, especially the hepatocellular carcinoma (HCC) cell lines. Therefore, the mechanism of action of PPD in HCC cells was elucidated. PPD inhibited the proliferation, migration, and invasion ability of HepG2 and PLC/PRF/5 cells in a dose-dependent manner. Western blot and immunofluorescence assay showed that PPD can alter the expression of epithelial–mesenchymal transition markers, increase E-cadherin expression, and decrease vimentin expression. Docking and biacore experiments revealed that STAT3 is the target protein of PPD, which formed hydrogen bonds with Gly583/Leu608/Tyr674 at the SH2 domain of STAT3. PPD inhibited the phosphorylation of STAT3 and its translocation from the cytosol to the nucleus, thereby inhibiting the expression of Twist1. PPD also inhibited tumor volume and tumor lung metastasis in PLC/PRF/5 xenograft model. In conclusion, PPD can inhibit the proliferation and metastasis of HCC cells through the STAT3/Twist1 pathway.

## Introduction

Hepatocellular carcinoma (HCC) is the third leading cause of cancer-related death worldwide due to the lack of effective therapy options. Tumor recurrence and metastasis are common in HCC^1^. Although many target drugs and treatment methods have been developed, no therapeutic drug can effectively cure liver cancer^2,3^. The epithelial–mesenchymal transition (EMT) process is associated with dramatic changes in cellular morphology and has been considered a mechanism that leads to tumor metastasis^4^. The EMT plays a crucial role in human liver cancer and is associated with tumor invasion, intrahepatic metastasis, and poor prognosis. The molecular mechanisms of hepatocyte EMT have been confirmed^5–8^.

Rare ginsenosides extracted from ginseng have substantial biological activities, including immune regulatory, antioxidation, anti-inflammation, and antitumor effects^9,10^. These ginsenosides are mainly divided into diol- and triol-type ginsenosides. To date, 100 types of ginsenoside compounds have been identified^11^. Diol-type ginsenosides mainly include Rb1, Rd, Rh2, Rg3, Rg5, and Rk1^12^. Several studies reported the antitumor effects and mechanisms of different diol-type ginsenoside-related pathways, including ROS/JNK/p53, Wnt/β-catenin, and ERK. Protopanaxadiol (PPD) is a diol-type ginsenoside. Unfortunately, reports on the antitumor effects of PPD and its molecular mechanisms are insufficient^13–16^.

In this study, the antitumor effects of five diol-type ginsenoside monomers in five different cancer cell lines were evaluated. Results indicated that PPD exhibits excellent antitumor effects on HCC cells, and thus the antitumor mechanisms of PPD on HCC were evaluated.

## Materials and methods

### Materials

Ginsenoside was purchased from Enoch Phytomedicine Ltd. (Shenzhen, China). Crystal violet and 3-(4,5-dimethylthiazol-2-y1)-2,5-diphenyltetrazolium bromide (MTT) were purchased from Sangon Biotech (Shanghai, China). Matrigel and transwell chambers were purchased from BD Biosciences (San Jose, CA, USA). Lipofectamine 2000 (Invitrogen, USA).

### Cell culture

The following human cell lines were obtained from KeyGen Biotech (Nanjing, China): liver (PLC/PRF/5), lung (NCI-A549), breast (MCF-7), and colon (HCT-8) cancer cell lines. The cells were grown in 1640 medium supplemented with 10% fetal bovine serum (Hyclone, USA) and maintained at 37 °C with 5% CO_2_ in a humidified atmosphere. The liver (HepG2) and pancreatic (PANC-1) cancer cell lines were cultured in DMEM medium supplemented with 10% fetal bovine serum (Hyclone, USA) and maintained at 37 °C with 5% CO_2_ in a humidified atmosphere. The siRNA of STAT3 was obtained from GenePharma Technology (Shanghai, China). The siRNAs of STAT3 and siRNA control vector were transfected into PLC/PRF/5 and HepG2 cells with Lipofectamine 2000 (Invitrogen, USA).

### Cell viability assay

The cells were plated in 96-well plates (4–6 × 10^3^/well) and treated with PPD, Rg5, Rh2, Rh3, and Rg3 for 48 h at 37 °C in a humidified atmosphere containing 5% CO_2_. The maximum concentration of the drugs was 210 µM, the equilibration gradient was 30 µM, and cell viability was determined by the standard MTT assay. Then, the culture medium was removed, and the cells were lysed using DMSO. Finally, the optical density values of the solution were determined at 562 nm with a microplate reader (Multiskan™ FC, Thermo Scientific). The value of IC50 was calculated by using GraphPad Prism7 software, and the mean ± standard deviation (SD) was obtained from three independent experiments.

### Wound healing assay

The PLC/PRF/5, NCI-A549, HCT-8, PANC-1, and MCF-7 cells were grown on 48-well plates to 100% confluence. The 100 µm wounds were scratched using sterile pipette tips. The PPD (20 and 40 µM) were added to cells cultured in medium (2% serum) for 48 h. The images of the cells were acquired with a light microscope (Nikon, Japan).

### Cell invasion assay

The transwell chamber used for the cell invasion assay was a polyethylene terephthalate filter containing 8.0 µm pores in a 24-well plate (Corning, USA). The filter was coated with Matrigel. Cells suspended in 200 µL of serum-free medium (1 × 10^5^ cells/mL) were seeded in the upper compartment of the transwell chamber, and the lower chamber is filled with medium containing 10% fetal bovine serum. Then, cells were treated with PPD (20 and 40 µM) for 24 h. Subsequently, the residue was wiped with a cotton swab. The cells were stained with 0.1% crystal violet for 10 min. Invading cells were visualized and counted in five randomly selected fields under an inverted microscope.

### Scanning electron microscopy (SEM)

The PLC/PRF/5 and HepG2 cells were treated with different concentrations of PPD for 48 h in a 24-well plate. After 48 h, the cells were fixed in precooled 2.5% glutaraldehyde at 4 °C for 2 h and 1% osmic acid at 4 °C for 1 h and then washed twice with PBS. The samples were dehydrated with different concentrations of ethanol and tert-butanol and then dried with a vacuum dryer. After vacuum freeze­-drying, the cells were coated with gold and photographed with a scanning electron microscopy (JEOL 6000).

### Colony formation assay

A suspension of PLC/PRF/5 and HepG2 cells were seeded into 6-well plates (500 cells/well) and incubated for 14 days. The 6-well plates were fixed with 4% paraformaldehyde for 20 min. The medium was discarded, and the cells were carefully washed twice with PBS and then stained with 0.01% crystal violet. Then, the number of colonies was counted under the microscope.

### Immunofluorescent staining

The PLC/PRF/5 and HepG2 cells with different treatments were washed three times with 1× PBS, fixed in 4% paraformaldehyde (precooled at 4 °C, Solarbio) for 20 min, and blocked with 5% bovine serum albumin (BSA, KeyGen Biotech) containing 0.1% Triton X-100 (Sigma) for 30 min at room temperature. Then, the resultants were incubated with anti-E-cadherin, anti-vimentin, and anti-STAT3 (1:100, Affinity). The cells were washed with 1× PBS again and subsequently incubated with fluorescently conjugated secondary antibodies (1:200, KeyGen Biotech) diluted in 5% BSA for approximately 50 min at room temperature. Finally, the cells were washed with 1× PBS and mounted with the DAPI-containing mounting medium (Solarbio). Images were obtained with a laser scanning confocal microscope (Nikon, Japan).

### Molecular docking

The 3D structures of the PPD were generated with LigPrep and minimized with optimized potentials for liquid simulations OPLS-2005 force field by Schrodinger software. The STAT3 dimer was obtained from the Protein Data Bank. The protein structure was prepared by assigning bond orders, particularly by adding hydrogen, optimizing H-bond assignment, and relaxing the structure through energy minimization with OPLS-2005 force field in a vacuum. The ligand of the crystal structure was used to define the central site of the docking grid box, and the *xyz* dimensions of the docking grid box were set to 60 × 60 × 60.

### Biacore

The experiment used the control software version 3.0 and Sensor Chip CM5 (carboxymethylated dextran surface), and the experiments were conducted at 25 °C by using a Biacore S200 SPR sensor (Biacore). Ginsenoside PPD was injected into different concentrations of protein and blank channels (0–25 μM), and the supernatant flow rate was 20/min. The protein binding period was set to 3 min, and the decomposition period was adjusted to 30 s.

### Dual-luciferase assay

Dual-reporter constructs were transfected into the PLC/PRF/5 and HepG2 cells by using transfection reagents. After changing to fresh medium 24 h after transfection, the cells were treated with various concentrations of PPD. The culture medium was collected into a 96-well white plate after 48 h, and luminescence was measured with a luminometer.

### Western blot analysis

Proteins were extracted from the PLC/PRF/5 and HepG2 cells treated with different drugs and analyzed through western blot analysis. The proteins were incubated with primary antibodies against GAPDH, E-cadherin, vimentin, and p-STAT3 (Affinity, 1:1000). The blots were further incubated with HRP-labeled secondary antibodies (Affinity, 1:5000). Finally, the target proteins were visualized by using ECL substrate reagents (Millipore, USA).

### Proteomics analysis and survival analysis

The PLC/PRF/5 cells were cultured in a 6-well plate. After 48 h, samples from the control group and PPD-treated group were collected for proteomic analysis. The differentially expressed proteins, which were significantly regulated (|logFC| > 2) in the PPD-treated samples, were analyzed through Gene Ontology (GO) and Kyoto Encyclopedia of Genes and Genomes (KEGG). The protein–protein interaction (PPI) network was analyzed with the STRING website (www.string-db.org/) and Cytoscape software. To obtain reliable data, we only selected the interactions with combination scores of >0.9. To determine which protein play substantial roles in the PPI network, we used CentiScape 2.2 plug-in module of Cytoscape to calculate the degree of connectivity in the PPI network.

Kaplan–Meier curve showing the 10-year survival of samples was obtained from TCGA (https://portal.gdc.cancer.gov/). LIHC samples were classified by STAT3 or Twist1 expression level (https://www.proteinatlas.org/).

### Animal studies

Female BALB/C nude mice (5–6 weeks old) were maintained in animal care facilities without specific pathogens. All the animal studies were conducted in accordance with the National Institutes of Health Animal Use Guidelines and current Chinese Regulations and Standards for the Use of Laboratory Animals. All the animal procedures were approved in accordance with the guidelines of the Animal Ethics Committee of the Tianjin International Joint Academy of Biotechnology and Medicine. The PLC/PRF/5 xenografts of tumors (1 × 10^6^/mL) suspended in PBS were established by a subcutaneous injection into the flank. The mice were randomly divided into four groups (*n* = 5), namely, control, PPD (30 mg/kg), OXA (10 mg/kg), and OXA + DDP (30 mg/kg DDP + 10 mg/kg OXA), after the tumors reached a volume of approximately 100 mm^3^ and then intragastrically administered every day. Tumor diameter and body weight were measured every other day during dosing, and tumor volume was calculated according to the formula *V* = *ab*^2^/2 (*a* = tumor length, *b* = tumor width). The administration time was continued for approximately 6 weeks. All the mice were euthanized, and xenografts were excised. All the tumors and lungs were fixed in 10% formalin for subsequent experiments.

### IHC assay and analysis

Tissues were deparaffinized and rehydrated through incubation with xylene and decreasing concentrations of ethanol. Endogenous peroxidase activity was blocked with 2% hydrogen peroxide. These microwave samples were incubated overnight with primary antibodies at 4 °C after blocking with rabbit polyclonal anti-E-cadherin and anti-vimentin. The E-cadherin and vimentin antibodies were diluted to 1:100. Rabbit polyclonal brown-stained cytoplasm, nuclei, or membranes in the cells were considered positive. Staining intensity was scored as follows: none (0), weak brown (1+), moderate brown (2+), and strong brown (3+). The percentages of the positive cells were divided into five classes on the basis of the percentage of tumor cells stained: 0 for no cells, 1 for 1–25%, 2 for 25–50%, 3 for 50–75%, and 4 for >75%.

### Statistical analysis

Three independent experiments using the PLC/PRF/5 and HepG2 cells were performed. Statistical analyses were performed with GraphPad Prism. One-way analysis of variance was conducted on the data in each group. SPSS24.0 was used. Measurement data were expressed as means ± SD (X ± SD). A difference of *P* < 0.05 was considered statistically significant.

## Results

### Diol-type ginsenosides inhibit cancer cells proliferation

**Fig. 1:**
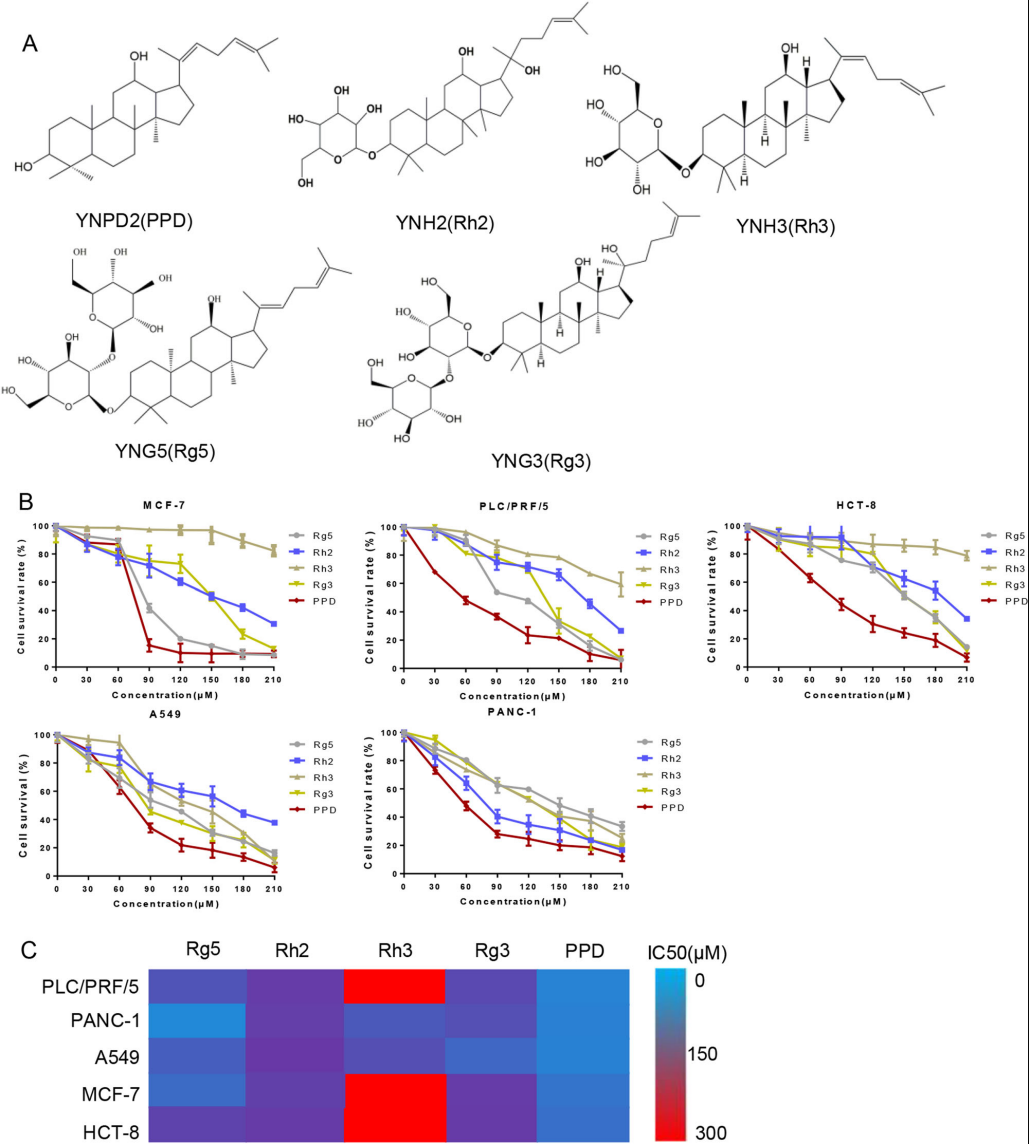
Effect of diol-type ginsenosides on the cell viability of cancer cells. **a** Chemical structure of diol-type ginsenosides used in this study. **b** Inhibitory effects of diol-type ginsenosides on the cell viability of PLC/PRF/5, MCF-7, A549, PANC-1, and HCT-8 cells were analyzed by MTT assay. **c** Summary of IC50 values for each group. PPD has an excellent inhibitory effect on the viability of cancer cells. Data are presented as means of three experiments, and error bars represent SD (*P < 0.05 and **P < 0.01)

We selected five kinds of monomers, namely, Rg5, Rh2, Rh3, Rg3, and PPD to verify the ability of diol-type ginsenosides in exhibiting inhibitory effects on cell viability (Fig. 1a). The antitumor effects of the ginsenosides were detected for 48 h on five different cancer cell lines (PLC/PRF/5, PANC-1, A549, MCF-7, and HCT-8) with various concentrations. As shown in the cell survival curves, the IC50 of PPD for each cell line is approximately 70 µM, which is lower than the values of the other derivatives (Fig. 1b). Among the five diol-type ginsenosides, PPD exerted the best inhibitory effect on the cell viability of each cell line (Fig. 1c).

### Diol-type ginsenosides inhibit the migration ability of cancer cells

**Fig. 2:**
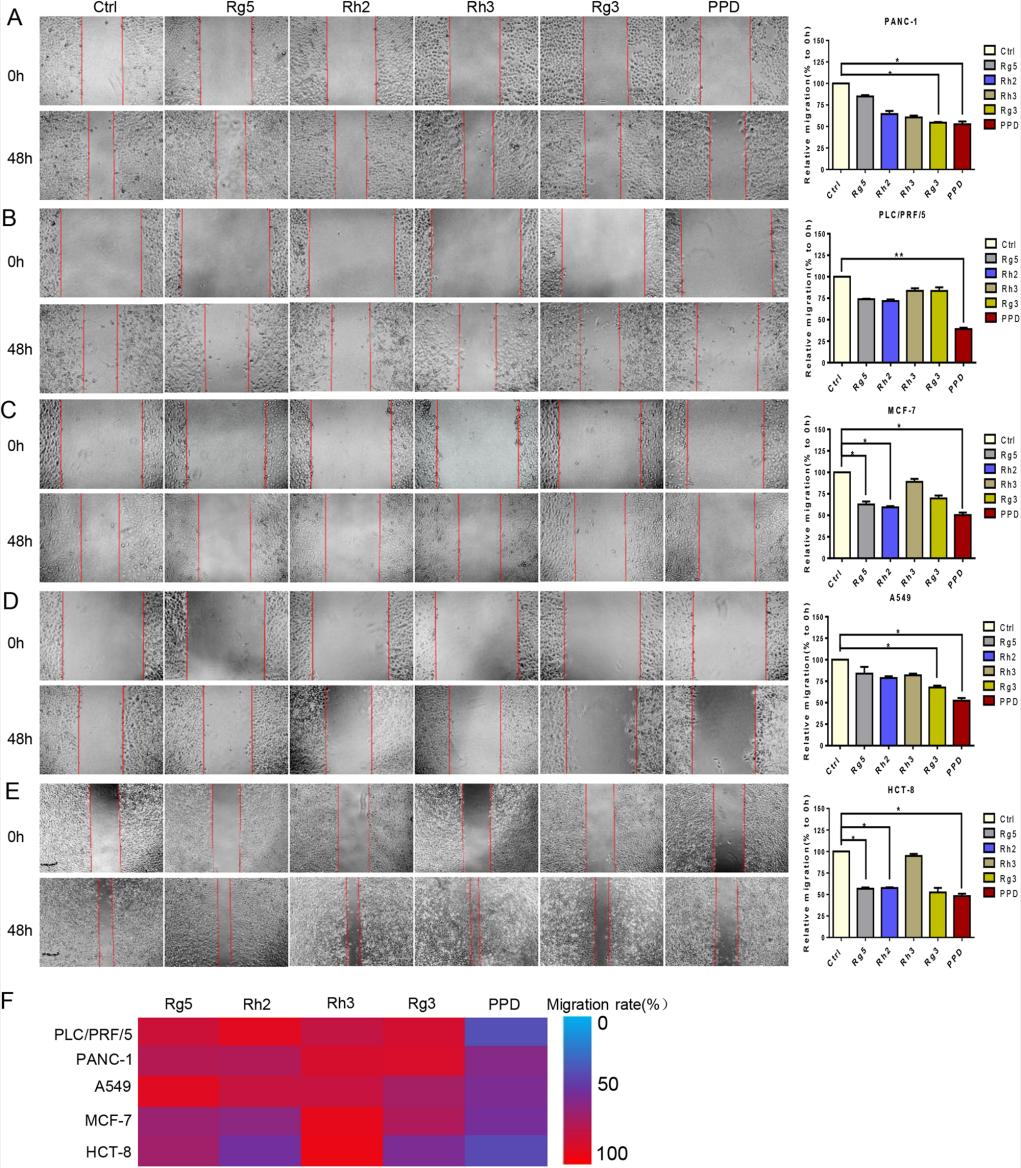
Effect of diol-type ginsenosides on the migration ability of cancer cells. **a** Effects of five diol-type ginsenosides on the migration ability of PLC/PRF/5 cells. **b** Effects of five diol-type ginsenosides on the migration of PANC-1 cells. **c** Effects of five diol-type ginsenosides on the migration of MCF-7 cells. **d** Effects of five diol-type ginsenosides on the migration of A549 cells. **e** Effects of five diol-type ginsenosides on the migration of HCT-8 cells. **f** Summary of migration rate for each group. PPD showed excellent inhibitory effect on the migration ability of cancer cells

We performed a wound-healing assay to investigate the ability of diol-type ginsenosides in inhibiting cancer cell migration. Ginsenoside PPD showed an inhibitory effect on the migration of the PANC-1, PLC/PRF/5, A549, MCF-7, and HCT-8 cells. However, PPD exhibited notable migration inhibition (i.e., 37%) in the PLC/PRF/5 cells (Fig. 2a–e). PPD had the best inhibitory effect on the migration of each cell line (Fig. 2f) and had the best inhibitory effect on the invasion of the PLC/PRF/5 cells (Fig. S1).

### PPD inhibits the migration, invasion, and EMT of HCC cells

**Fig. 3:**
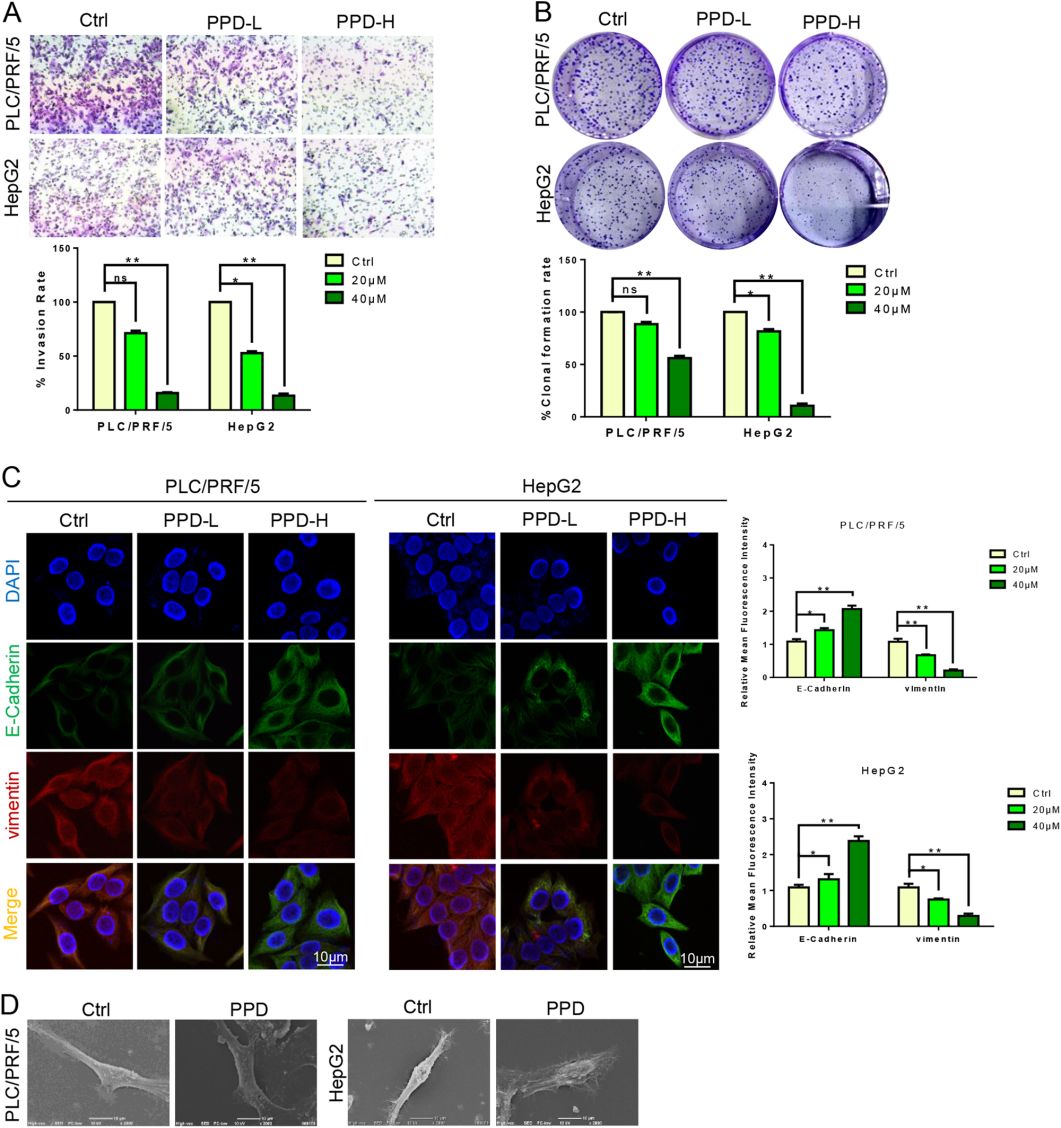
PPD inhibited the invasion and EMT of the PLC/PRF/5 and HepG2 cells. **a** PPD inhibited the invasion ability of PLC/PRF/5 and HepG2 cells. **b** PPD inhibits the colony forming ability of hepatoma cell lines. **c** Typical images of immunofluorescent double staining for E-cadherin and vimentin in PLC/PRF/5 and HepG2 cells treated with PPD. **d** PPD changes the morphology of PLC/PRF/5 and HepG2 cells. Data are presented as means of three experiments, and error bars represent SD (*P < 0.05 and **P < 0.01)

The HCC still has no available effective treatment despite being a malignant tumor. Meanwhile, PPD shows an excellent therapeutic effect on HCC cancer. The effect of PPD on the EMT of HCC cells was also studied. Matrigel-coated transwell chambers were used for the testing of the invasion ability of the PLC/PRF/5 and HepG2 cells after administration and investigating the ability of PPD to inhibit the invasion of both cell lines. The PPD inhibited the invasive ability of PLC/PRF/5 and HepG2 cells in a dose-dependent manner (Fig. 3a). Then, the effect of PPD on the cloning ability of HCC cells was explored. The results of colony formation showed that the cell cloning ability was inhibited in a dose-dependent manner (Fig. 3b). E-cadherin and vimentin are EMT markers. The PLC/PRF/5 and HepG2 cells were treated with different doses of PPD for 24 h. Subsequently, E-cadherin and vimentin were immunofluorescent double stained to observe the fluorescence intensity. The expression levels of vimentin were decreased and E-cadherin were increased by PPD (Fig. 3c). We also detected the expression level of EMT-related marker VE-cadherin. PPD can inhibit the expression of VE-cadherin dose-dependently (Fig. S2). The morphological changes were further characterized with optical and scanning electron microscopes. Low doses of PPD led to the extension of pseudopodia and changes in microfilament structure (Fig. 3d).

### PPD targets STAT3 and inhibits Twist1 expression

**Fig. 4:**
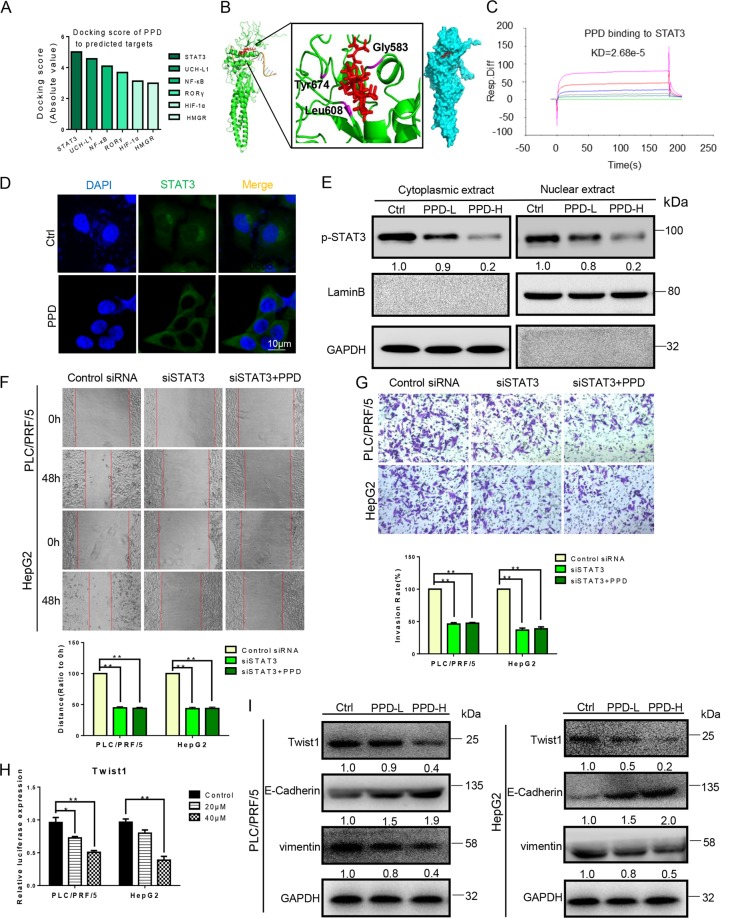
PPD inhibited EMT of HCC cells by targeting STAT3. **a** Molecular docking scores for PPD and six potential targets. **b** Predicted model of PPD binding to the SH2 domain of STAT3 as shown by computational docking. The protein backbone is portrayed as a transparent green ribbon, and PPD is depicted as atom-red sticks-and-balls. **c** Biacore analysis results of PPD and STAT3. **d**, **e** PPD inhibited the translocation of STAT3 to the nucleus in PLC/PRF/5 cells detected by immunofluorescence and Western blot. **f**, **g** Effect of PPD on the migration and invasion of STAT3 knockdown PLC/PRF/5 and HepG2 cells. **h** Dual-luciferase assay results for PLC/PRF/5 and HepG2 cells suggest that Twist1 is a target gene of PPD. **i** Protein expression level changes of Twist1, E-cadherin, and vimentin in the PLC/PRF/5 and HepG2 cells treated with PPD. The GAPDH blot served as a loading control

The mechanism of PPD inhibition of EMT was explored on the basis of the potential target predicted on the website (http://sea.bkslab.org/). Molecular docking was performed, and the docking score was calculated by Schrodinger software. The predicted STAT3 targets were docked with PPD, and the target with a high docking score was selected (Fig. 4a). The active sites of PPD and STAT3 dimer (protein structure information was obtained from protein data bank entry 1BG1) were examined by combining points through molecular docking. PPD formed hydrogen bonds with Gly583/Leu608/Tyr674 at the SH2 domain of STAT3 (Fig. 4b). We then performed a biacore experiment. PPD directly binds to STAT3 in a concentration-dependent manner and has micromolar binding affinity (KD = 2.68e−5 M; Fig. 4c). STAT3 can be involved in the regulation of apoptosis, migration, invasion, and angiogenesis. We further used immunofluorescence and western blot experiments to verify the ability of PPD to affect STAT3 nuclear translocation, considering that nuclear translocation is the core of transcription factor function. In the PPD treatment, STAT3 was inhibited from shifting to the nucleus of the HCC cells (PLC/PRF/5; Fig. 4d). In the western blot experiment, PLC/PRF/5 cell was exposed to PPD for 48 h. Nuclear and cytoplasmic extracts of PPD-treated or untreated cells were prepared with a Nuclear and Cytoplasmic Extraction Reagent Kit. After the PPD treatment, the nuclear expression of p-STAT3 decreased in a concentration-dependent manner (Fig. 4e). To verify the effect of PPD on STAT3 pathway, we further determined the effects of PPD on the migration and invasion of STAT3 knockdown PLC/PRF/5 and HepG2 cells. Wound-healing and invasion experiments showed that the migration and invasion ability were decreased in STAT3 knockdown PLC/PRF/5 and HepG2 cells. PPD had no significant effect on the migration and invasion ability of STAT3 knockdown PLC/PRF/5 and HepG2 cells (Fig. 4f, g). The constitutive activation of STAT3 promoted the expression of Twist1. Thus, we tested the expression of Twist1 in the PLC/PRF/5 and HepG2 cells. The dual-luciferase assay results showed that PPD inhibited Twist1 expression (Fig. 4h). Western blot results showed that the expression levels of vimentin and E-cadherin decreased and increased, respectively. Meanwhile, Twist1 expression was diminished, and thus EMT was inhibited (Fig. 4i).

### PPD enhances the antitumor effects of oxaliplatin in a mouse xenograft model and alters EMT marker levels in cancer tissues

**Fig. 5:**
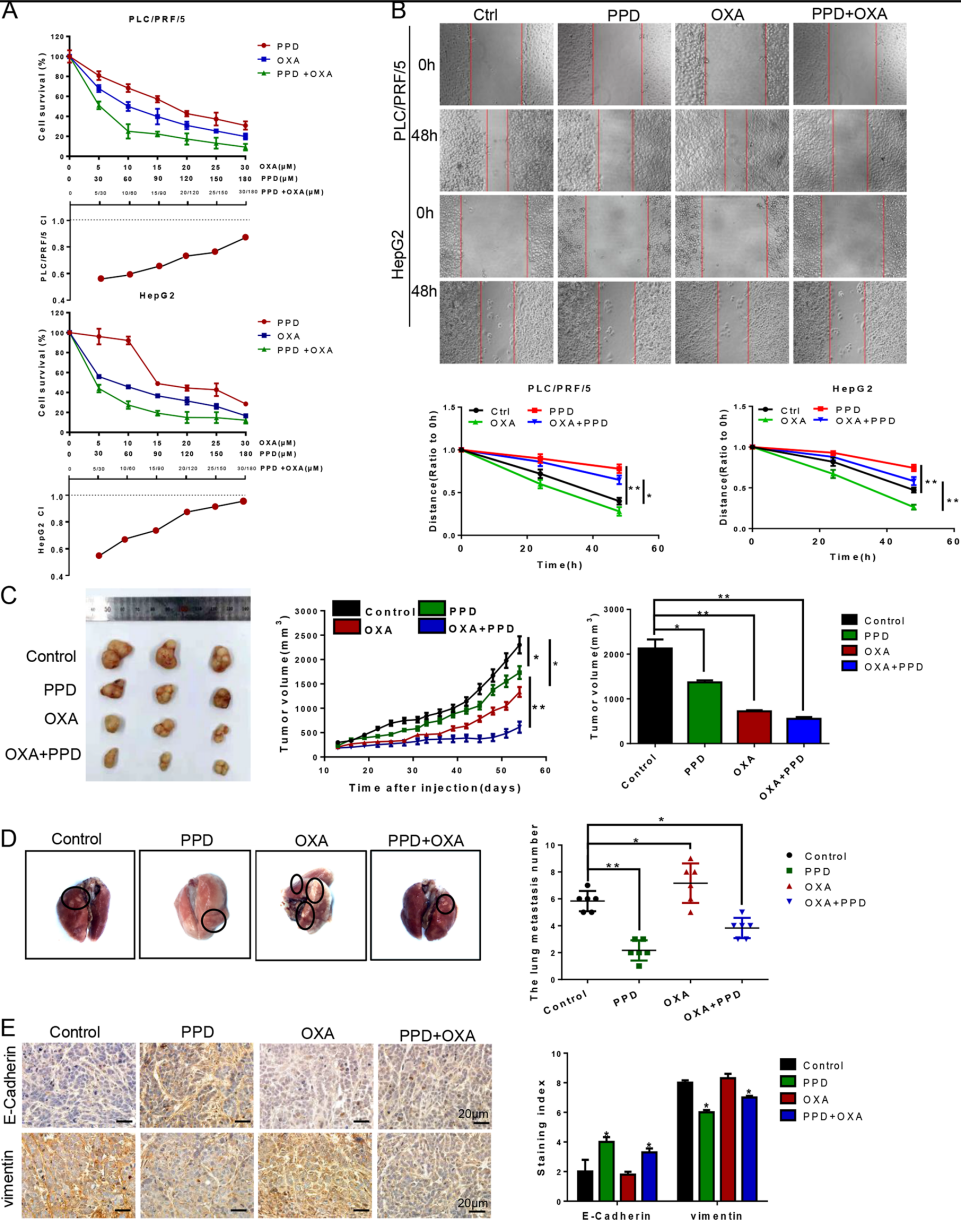
Effect of PPD on a nude mouse PLC/PRF/5 xenograft model. **a** Determination of the sensitizing effect and CI value of PPD and oxaliplatin by MTT assay. **b** Wound healing assay of PLC/PRF/5 and HepG2 cells treated with PPD, OXA, or a combination of PPD and OXA. Wound healing was observed 48 h after treatment. **c** Changes in the tumor volumes of the PLC/PRF/5 xenografts. Tumor growth was considerably suppressed in the combined treatment group compared with the tumor growths in the PPD and OXA groups. **d** Number of tumors that shifted to the lungs in different groups. PPD considerably inhibited lung metastasis. **e** Immunohistochemical staining for E-cadherin and vimentin in tumor samples from xenografts. Data are presented as means of three experiments, and error bars represent SD (*P < 0.05 and **P < 0.01)

Oxaliplatin (OXA) is a third-generation platinum anticancer drug. Meanwhile, OXA-based chemotherapy is considered to be an important treatment choice for advanced-stage HCC^17^. We determined the effect of 48 h treatments using PPD, OXA, or both with an MTT assay. PPD and OXA diminished cell viability in a dose-dependent manner. PPD enhanced the inhibitory effects of OXA on HCC cell proliferation. Compusyn software analysis showed that the combination index (CI) value of the combined treatment group was <1 at different doses, that is, a synergistic effect is present between PPD and OXA (Fig. 5a). In the wound healing assay, the migration ability of cells treated with OXA alone increased after 24 and 48 h of treatment, thereby indicating that OXA promotes the migration of HCC cells. The wound gap was wide in the combined treatment groups. Thus, the combined treatment inhibited the motility of HepG2 and PLC/PRF/5 cells (Fig. 5b). The PLC/PRF/5 cells were seeded into BALB/C nude mice for the investigation of the in vivo antitumor effect of PPD. Tumor growth was slightly suppressed in the PPD and OXA groups compared with the control group and considerably suppressed in the co-treatment group. These results strongly suggested that PPD can enhance the antitumor effect of OXA (Fig. 5c). The number of tumors that shifted stoves decreased in the lungs of nude mice with PLC/PRF/5 xenografts treated with PPD compared with those of nude mice in the other groups (Fig. 5d). Immunohistochemical staining of tumor tissues of each group showed that the treatment of PPD increased and decreased the expression levels of E-cadherin and vimentin, respectively, relative to the levels in the control group (Fig. 5e).

### Effects of PPD on proteomics profiles of HCC cells

**Fig. 6:**
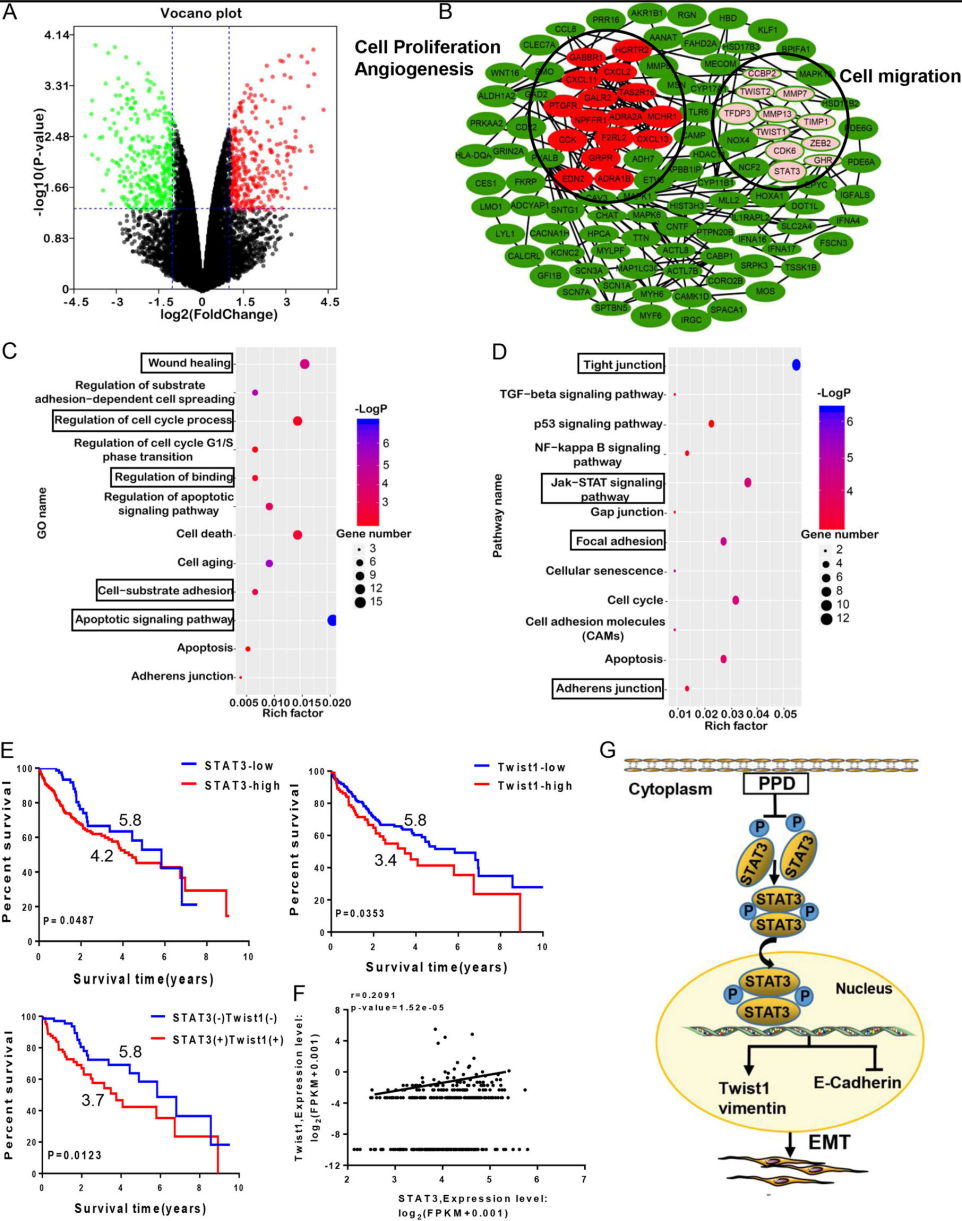
Bioinformatic analysis of PPD and survival analysis of key proteins in HCC. **a** Volcano plot of the differentially expressed proteins between PPD-treated and untreated cancer cells. Red and green dots indicate the up- and downregulation of proteins, respectively. **b** Protein interaction network of PPD. **c** GO analysis of differentially expressed proteins. **d** KEGG analysis of differentially expressed proteins. **e** Kaplan–Meier curve showing the 10-year survival of TCGA LIHC samples classified by STAT3 or Twist1 expression. The correlation coefficient and *P* value are shown. **f** Correlation analysis of STAT3 with Twist1 in TCGA LIHC samples. The correlation coefficient and *P* value are shown. **g** Schematic diagram of PPD blockage possible mechanism in EMT via targeting STAT3/Twist1 signaling

The volcano map was used to macroscopically express the up- or downregulation of proteins by PPD. After the PPD treatment, 110 and 120 proteins were up- and down-regulated, respectively (Fig. 6a). The PPI network showed that the functions of the subnetworks (selected by M-CODE) are mainly associated with cell migration and proliferation and angiogenesis (Fig. 6b). The GO analysis results showed that the differentially expressed proteins were enriched in the functions of wound healing, apoptotic signaling pathway, regulation of cell cycle process and binding, and cell-substrate adhesion (Fig. 6c). The KEGG pathway analysis revealed that the differential proteins were mainly involved in several pathways, including tight junction, p53, JAK-STAT signaling pathways, focal adhesion, and cell cycle (Fig. 6d). We analyzed the effect of Twist1 and STAT3 expression on survival status on the basis of the TCGA database. Survival analysis revealed that the high expression of STAT3 or Twist1 indicates worse prognosis (Fig. 6e). Correlation analysis of STAT3 with Twist1 was also conducted, and the results showed that the expression levels of STAT3 and Twist1 are positively correlated. The STAT3/Twist1 axis promoted the malignant progression of patients with HCC. Therefore, the STAT3/Twist1 axis may be an effective target for HCC therapies (Fig. 6f).

## Discussion

We evaluated the antitumor effects of diol-type ginsenosides in different tumor cell lines. PPD showed excellent antitumor effect in HCC and inhibited the migration, invasion, and proliferation of HCC cells in a dose-dependent manner. PPD also inhibited the occurrence of EMT. Further experiments showed that PPD can target STAT3 and play a role in antitumor activity.

Tumor recurrence and metastasis are common in HCC^18^. EMT is generally considered to be the core process of tumor metastasis^19^. During this process, cells lose their epithelial characteristics and acquire mesenchymal cell properties and subsequently show high metastatic potential^20^. E-cadherin and vimentin mediate cell adhesion and are two EMT biomarkers^21,22^. In the present study, the expression levels of E-cadherin and vimentin in both hepatoma cell lines (PLC/PRF/5 and HepG2) were examined. PPD effectively inhibited the EMT process in HCC and increased the expression level of E-cadherin and decreased that of vimentin.

We further explored the molecular mechanism of PPD affecting EMT. STAT3 was proven to be the target of PPD on the basis of the target prediction and docking result of PPD. STAT3 has a pivotal role in multiple oncogenic processes, such as proliferation, migration, survival, and angiogenesis^23^. Generally, STATs are localized in the cytoplasm in an inactive state. When stimulated by cytokines, STAT3 binds to DNA after entering the nucleus and then participates in the regulation of cell migration, invasion, and angiogenesis^24–26^. STAT3 inhibition strategies include the inhibition of the STAT3 DNA binding domain, suppression of the STAT3 SH2 domain, and inhibition of the STAT3–importin interaction^27,28^. The SH2 is a highly conserved domain of the family and is required for the formation of STAT3 dimers upon phosphorylation. Targeting the SH2 domain can inhibit the expression of p-STAT3 at Tyr-705 but does not affect the expression of p-STAT3 at Ser727 and total STAT3^29,30^. PPD can target the SH2 domain and diminish the tyrosine phosphorylation of STAT3 (p-STAT3 at Tyr705) and subsequently affects the STAT3 dimerization and entry into the nucleus. PPD treatment did not inhibit the migration and invasion ability of the STAT3 knockdown cells. This result further indicated the effect of PPD on STAT3 pathway. Some studies showed that Twist1 is a highly important transcription factor in EMT, and the overexpression of Twist1 induces EMT, which is a key process in tumor metastasis^31,32^. In our research, we found that PPD inhibits the Twist1 expression and EMT by targeting the SH2 domain of STAT3 and translocation from the cytosol to the nucleus (Fig. 6g).

In conclusion, PPD can effectively inhibit cell proliferation, migration, invasion, and EMT through the STAT3-Twist1 signaling pathway. PPD can enhance the sensitivity of HCC cancer to oxaliplatin and considerably inhibit the lung metastasis of tumors. These results indicated that PPD may be a new antitumor candidate.

## Supplementary information

supplementary materials
