# Supplementary material for: Protopanaxadiol inhibits epithelial–mesenchymal transition of hepatocellular carcinoma by targeting STAT3 pathway
Source: Cell Death Dis. 2019 Aug 20;10(9):630. doi: 10.1038/s41419-019-1733-8 (PMC6702205; doi:10.1038/s41419-019-1733-8)
Supplement: Supplementary file 1 — supplementary materials [file 41419_2019_1733_MOESM1_ESM.docx]

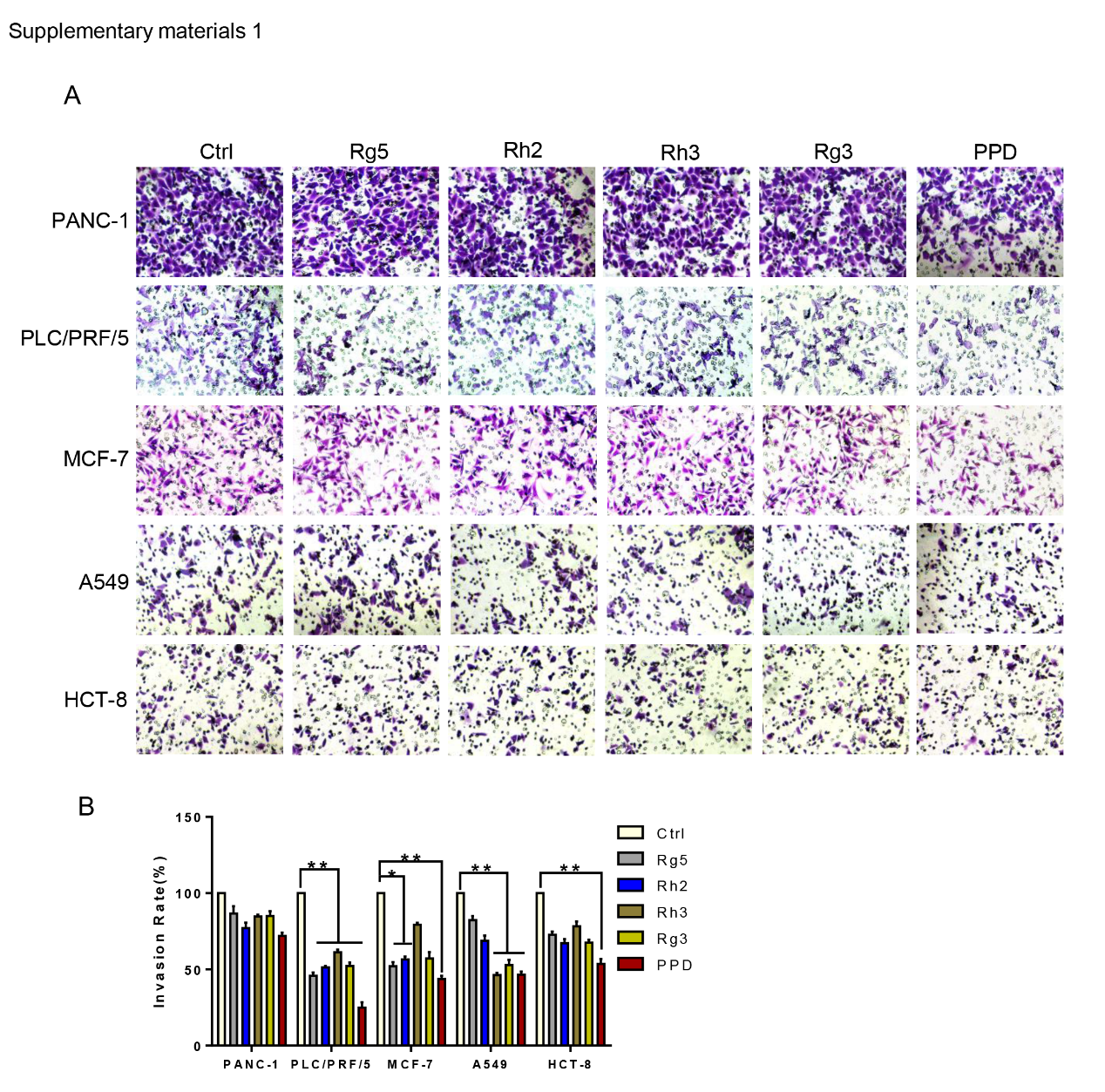


**Supplementary materials 1. Effect of diol-type ginsenosides on the invasion of cancer cells.** (A-B) Effects of five diol-type ginsenosides on the invasion ability of each cell line detected using transwell. Data are presented as means of three experiments, and error bars represent SD (*P < 0.05 and **P < 0.01).


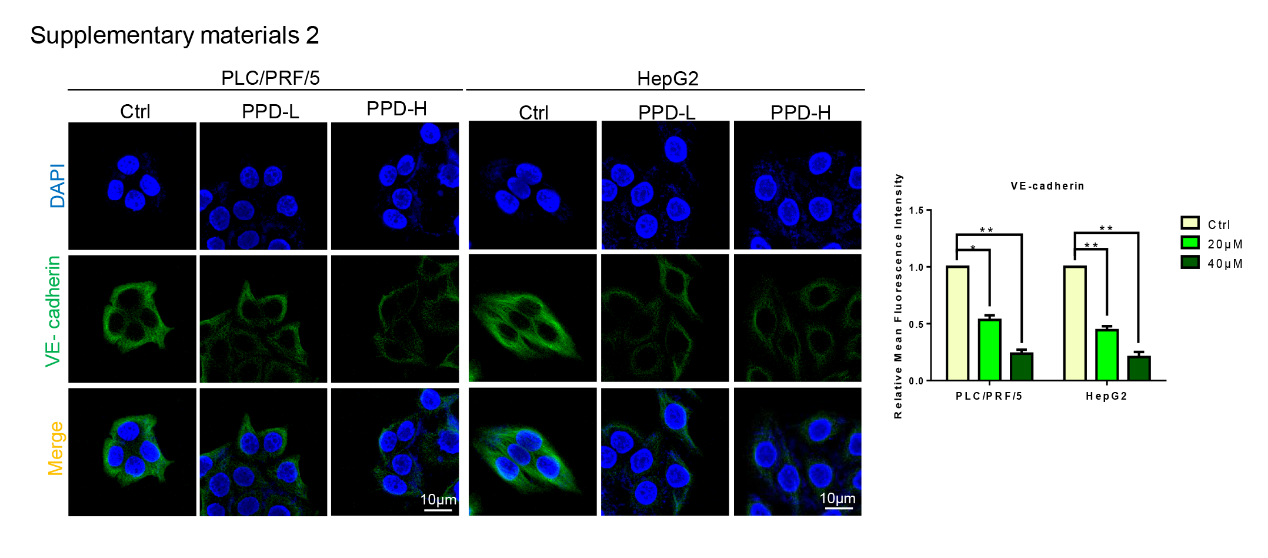


**Supplementary materials 2.** **PPD inhibited the expression of VE-cadherin in PLC/PRF/5 and HepG2 cells.** (A) Typical images of immunofluorescent staining for VE-cadherin in PLC/PRF/5 and HepG2 cells. Data are presented as means of three experiments, and error bars represent SD (*P < 0.05 and **P < 0.01).


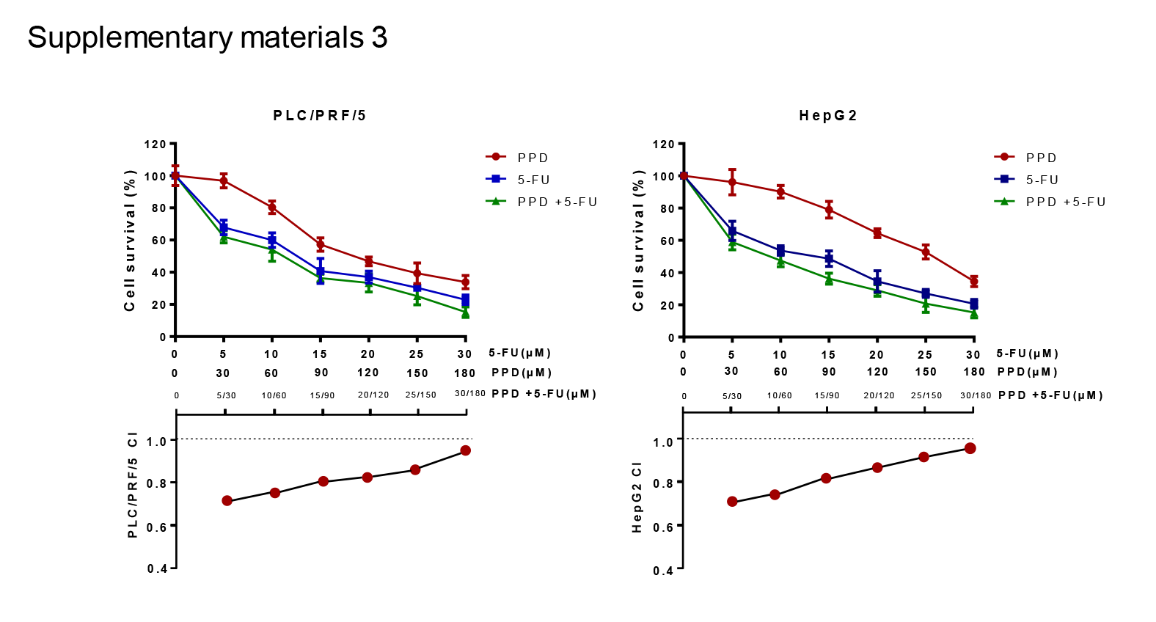


**Supplementary materials 3.** Determination of the sensitizing effect and CI value of PPD and 5-fluororacil by MTT assay.
